# Inverting the patient involvement paradigm: defining patient led research

**DOI:** 10.1186/s40900-018-0104-4

**Published:** 2018-07-10

**Authors:** Laura B. Mader, Tess Harris, Sabine Kläger, Ian B. Wilkinson, Thomas F. Hiemstra

**Affiliations:** 1 Cambridge Clinical Trials Unit, Cambridge University Hospitals NHS Foundation Trust, Cambridge, UK; 2 School of Clinical Medicine, University of Cambridge, Cambridge, UK; 3 Polycystic Kidney Disease Charity, London, UK

**Keywords:** Co-production, Patient, Public, Involvement, PPI, Research, Trials

## Abstract

**Plain English Summary:**

Patients usually understand their disease and lifestyle needs better than many medical professionals. They also have important ideas about what research would be most beneficial to their lives, especially on how to manage symptoms in a way that improves daily quality of life. In the UK, the National Institute for Health Research has recognised the value of patient insight, and now requires researchers with public funding to involve patients and the public throughout the research process. There are many opportunities for involvement, but these generally focus on improving study design to ensure the trial is acceptable to participants. Some programmes work towards setting research priorities as important to patients, public members, and medical experts, but due to the complexity and cost involved in running clinical trials, the majority of research originates with the pharmaceutical industry or academic institutions. There is a clear mismatch between research ideas that patients prioritise (quality of life), and those actually investigated (drug development).

The Patient Led Research Hub (PLRH) is a new initiative hosted by the Cambridge Clinical Trials Unit. The PLRH supports research ideas as proposed by patient organisations, providing resources and expertise in research design and delivery. The PLRH aims to co-produce any technically feasible project, regardless of disease or symptom focus. The proposing patient group maintains ownership of the project with an active role in study management. This method of research has proven to produce credible research studies that are of direct relevance to patients.

**Abstract:**

Patient and Public Involvement has become an indispensable and expected component of healthcare research in the United Kingdom, largely driven by the National Institute of Health Research and other research funders. Opportunities for patients to become involved in research abound, and many organisations now have dedicated ‘public involvement’ teams. However, its value is often questioned amidst criticism of tokenism and the recognition that a mismatch persists between patient priorities and funded research. Although patients are frequently consulted, evidence that their involvement influences the research agenda remains limited. We propose a novel model that allows patients and the public not only to propose research questions, but to design, initiate and deliver their own research with all the necessary support from research professionals. We demonstrate the feasibility and utility of this approach in reporting the establishment, experiences and progress of the Patient Led Research Hub. Using this resource, patient organisations are now able to initiate and conduct rigorous clinical research unfettered by the constraints of academic or economic agendas.

## The unmet need of patient led research

The design and delivery of clinical research and the setting of research agendas have long been the domain of academic researchers and industry. In industry, real or perceived clinical needs are identified, but priorities are almost invariably determined by economic considerations. Industry sponsored trials necessarily select interventions, comparators and trial outcomes in order to maximise the likelihood of success. Academic researchers may not be equally driven by commercial considerations, but instead are constrained by demands to produce work that is highly cited, of high impact, and most likely to result in further research funding. This environment lends itself to relatively narrow research programmes, often focused, topic specific and seldom diverging from a defined research narrative.

Patient involvement in research is a relatively recent development. The UK National Health Service launched the NHS Research and Development Strategy in 1991 [1], proposing patient involvement and engagement for the first time. This was formalised with the establishment of the ‘Standing Advisory Group on Consumer Involvement in the NHS Research and Development Programme’ in 1996, aiming to improve the way research is prioritised, commissioned, undertaken and disseminated [2]. Since then, there has been a rapid proliferation of initiatives and opportunities, with patient involvement now a prerequisite to research funding by the National Institute for Health Research (NIHR) and other grant organisations. Most funders ensure patient members serve on review panels and contribute to peer review. However, the overwhelming majority of commissioned and open research calls remain answered by academics or academic-industry partnerships. It is the norm for applicants to seek patient involvement only once research questions have been formulated and proposals are in draft. Notwithstanding the very real impact this approach has on the conduct of funded research, this situation may reasonably be described as a form of tokenism. The impact Patient and Public Involvement (PPI) activities have had on research outcomes continues to be questioned [3].

NIHR’s INVOLVE promotes PPI through a number of initiatives, provides tailored information and resources to patients and researchers, and hosts patient involvement events and conferences. A recent publication ‘Guidance on co-producing a research project’ aims to explain key principles and features important to realising research with patients and public as equal stakeholders [4]. The guidelines build on previous ‘Going the Extra Mile’ recommendations to promote co-production as a means of advancing traditional PPI, recognising the need for flexible, patient-centred opportunities [5].

Also supported by the NIHR, the James Lind Alliance (JLA) Priority Setting Partnerships have successfully engaged patients and stakeholder groups in setting top ten research priorities across a wide range of disease areas [6]. These partnerships and collaboration efforts are valuable, but many priorities thus set never proceed to trial, and those that do are commissioned with limited opportunity for continued patient and stakeholder involvement [7]. Many charities, patient support organisations, and disease-specific medical departments are also beginning to support internal patient priority setting. The National Cancer Research Institute and the Service User Research Enterprise, for example, provide an encompassing approach to cancer [8] and mental health research [9], by encouraging patients and service users to be involved across all aspects of research. However despite these efforts, a mismatch exists between patient priorities and interventions researched in registered trials [10], consistent with the observation that the research agenda of industry, academics and patients only partially overlap [7].

In the United States, PCORI is focused on patient centred outcomes, promoting patient involvement, and funding patient centred research, but proposals submitted by patients are internally prioritised and in turn commissioned externally [11]. In Canada, SPOR similarly focusses on patient-identified priorities and improved patient outcomes, achieved through a multi-disciplinary approach [12]. In Europe, organisations such as EUPATI focus on patient education [13]. The overall landscape of PPI initiatives are important and complementary, but do not yet allow patients to propose, initiate and conduct research in an independent manner. The net result is that many questions that matter a great deal to patients are never pursued or funded.

## Establishing the Patient Led Research Hub

In 2015, the UK Polycystic Kidney Disease (PKD) Charity approached members of the Cambridge Clinical Trials Unit (CCTU) to consider a trial assessing high water intake as a means of slowing polycystic kidney disease progression. Despite this question being of high importance to patients, the PKD Charity had been unable to collaborate with an academic partner since its first proposal in 2006. This experience highlighted the lack of resources and opportunities for patient groups with similarly unanswered questions, and led directly to the establishment of the Patient Led Research Hub (PLRH). PKD Charity and the PLRH have since successfully conducted an externally funded, randomised controlled feasibility trial (NCT02933268). Some of the feasibility work and the design of the trial are described in two joint publications [14, 15]. Following this success, a second proposal from the PKD Charity focused on chronic pain interventions in PKD; a high priority for patients but unlikely to receive industry funding. The PLRH, PKD Charity and 8 patients, initiated work on this project in 2017 with an international workshop to explore the topic and consider feasible trial designs.

The PLRH aims to provide academic expertise in all aspects of research design and conduct, free from a defined research agenda. This expertise is available to patients and patient organisations to support patient-initiated and patient led research. Positioned within the CCTU, with close ties to the University of Cambridge and Cambridge Biomedical Research Centre (BRC), core membership of the PLRH consists of an experienced clinical trialist and a full time research manager, with access to and input from statisticians, health economist, grants officer, and other expert members of the trials unit and School of Clinical Medicine. This core operational membership does not include patients or members of the public. Instead, oversight and input are sought from the Cambridge BRC PPI Oversight Group (which includes several members of the public), while patient members of the proposing group become part of the study management team for each individual PLRH project.

**Fig. 1:**
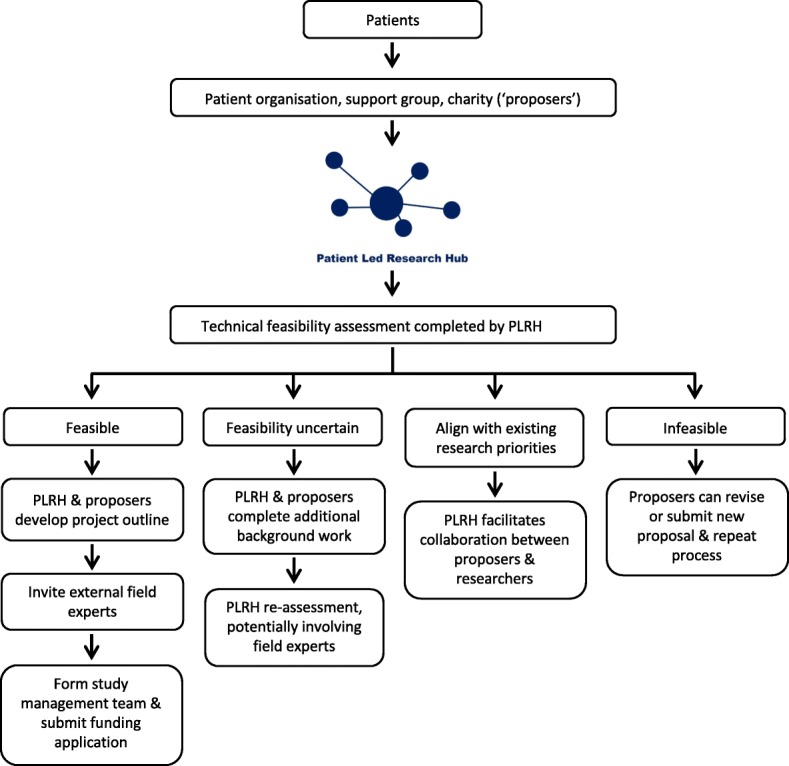
PLRH workflow

The PLRH was proposed to and supported by the Cambridge BRC and CCTU at its inception; this support includes funding for infrastructure and one full-time employee. Although independent of other local and regional PPI initiatives, interaction with existing PPI initiatives is maintained. The PLRH forms part of the Cambridge University Health Partners PPI Working Group, East of England Public Involvement in Research Partnership Group, and UK Clinical Research Collaboration (UKCRC) Patient Public Involvement and Engagement Task and Finish Working Group, ensuring support from experienced PPI Leads and access to public panels as needed. A process (Fig. 1) for accessing and for partnering with the PLRH was established in consultation with patient advisors, the Cambridge BRC PPI Oversight Group, and the CCTU Advisory Board. The PLRH and its activities are presented online, via social media, newsletters, and through direct contact with patient organisations.

## The PLRH patient involvement model

Research ideas or specific proposals are welcomed from any patient organisation or disease charity. Individual patient proposers are encouraged to first establish a dialogue with a relevant patient organisation before contacting the PLRH to foster a comprehensive support network and enable group collaboration. To ensure all proposals are considered equally without bias to patients experienced in research, there is no standardised form or set submission format. Proposals may be submitted online, via email or through direct conversation, and may be a simple idea or detailed project outline. Communication with the patient group ensures the proposal is thoroughly understood and translated into a viable research question as intended. The PLRH is not a priority setting organisation.

Tess Harris, patient and CEO of PKD Charity notes *“I was very impressed with the courtesy and professionalism shown to me in 2015 when we approached the PLRH. I was given time to explain why this was an important unanswered research question; further meetings were organised with relevant experts who gave time (pro bono) from their busy schedules to explore further; we were consulted and respected at every step; and listened as co-applicants/authors as relevant.”*

Once the research question is established, clinical trialists and statisticians conduct a preliminary assessment. The PLRH ethos is that, if a proposal is feasible and addresses an unmet need, it should be supported. The feasibility assessment considers only technical and operational criteria: 1) What is already known? 2) What sample size is required? 3) Can sufficient numbers of participants be recruited? 4) Is the proposed intervention available? 5) Is the research ethical? 6) Are there any potential funding sources? 7) What is the extent of clinician involvement required, and would the relevant clinicians support the research (for example, if medical or surgical interventions form part of the research, it may not be feasible without support from clinical staff)? If feasibility is uncertain, further work is undertaken by the PLRH and/or proposing group; this may include systematic reviews, patient surveys, or consultation with third parties including patient organisations, charities, funders, clinicians or academic researchers.

Decisions on feasibility are reached jointly with proposers. To date, there have been no instances where the feasibility decision has been disputed, but the PLRH makes clear that adjudication of any disputed decisions is possible through the Cambridge BRC PPI Oversight Group. However, given that the feasibility assessment is undertaken in partnership with proposers and that decisions are reached jointly, the likelihood of disagreement is small.

Infeasible proposals are not pursued, but the proposing group is welcome to revise or submit a new idea at any time. Feasible proposals that closely align with existing work are referred to the relevant research group or programme. Feasible projects supported by the PLRH require an ongoing partnership with the proposing patient organisation; proposals may be deferred if the proposing group is without capacity to lead the project. A collaborative study team is formed, and proposers maintain co-ownership of the emerging project, act as co-applicants on funding applications and all subsequent publications and retain intellectual property rights where appropriate. The PLRH project manager facilitates accessible and transparent dialogue throughout the lifespan of each project, ensuring equal representation of all stakeholders. Where large charities or patient organisations are involved, a variety of involvement and engagement opportunities are available to patient members throughout the project. External experts and national research groups are invited to contribute to project development. PPI activities and project development work are supported where possible through PLRH funds and external contributions (e.g. East of England Research Design Service (RDS)). This multifaceted approach enables competitive applications to public funders, research charities or industry partners. If funding is achieved, projects become autonomous to the extent possible to allow PLRH resources to become available for new proposals. Unsuccessful bids are reviewed in order to learn from these submissions and improve subsequent submissions. Retaining the ‘professional’ core PLRH membership allows the transfer of learning to any resubmission or similar future submissions in partnership with other groups. Based on our progress and experience to date, the PLRH intends to design a framework for future patient involvement in patient led research.

## PLRH progress: May 2015 – May 2018

**Fig. 2:**
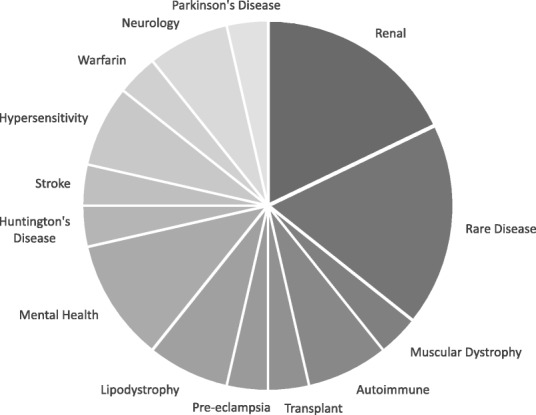
Primary topic area of all received proposals

The PLRH has received multiple high-quality research ideas and proposals, ranging in topic from bench to bedside (Fig. 2). Twenty-eight submissions were received between May 2015 and May 2018, originating from 14 different patient organisations and 11 individual patients living with a rare disease or mental health disorder. This number does not reflect all correspondence, as some organisations compile multiple proposals which are then prioritised based on importance to patient members and feasibility.

**Fig. 3:**
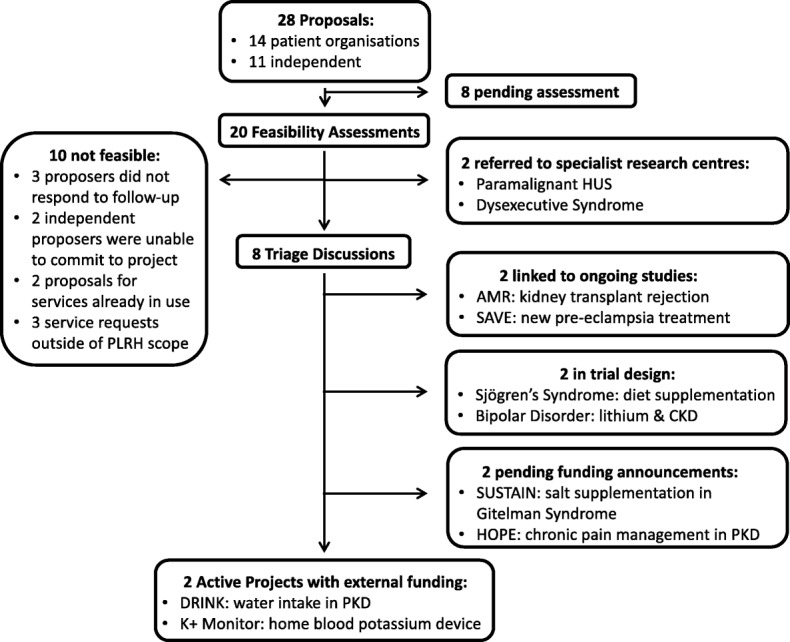
PLRH project status (May 2018)

Of submissions received, ten were not deemed technically feasible, were already answered in the literature, or proposers were unable to commit to the length of project. Two general research ideas that focused on raising awareness closely aligned with existing research programmes, and two proposals that focused on new treatments for disease modification mirrored clinical trials early in development; in each case the PLRH was able to facilitate collaboration between proposers and the relevant research group. Of the remaining 14 feasible proposals, two have become active projects, two are awaiting funding announcements, and ten are in varying stages of development (Fig. 3).

**Fig. 4:**
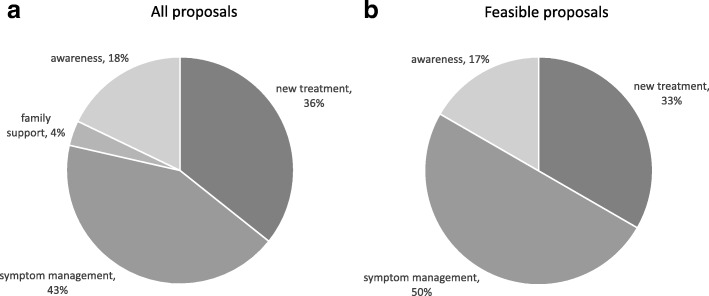
Primary outcome focus of **a** all patient proposals received, and **b** proposals deemed technically feasible. Symptom management to improve quality of life is the primary focus of proposals, followed by new treatment for disease modification, general information and raising awareness, and family/carer support

It has become clear from this activity that an unmet need exists to support patients’ own research proposals. Proposals received have had a striking focus on quality of life, with 50 % of feasible proposals concentrated on interventions to improve symptoms such as fatigue, pain, itch or depression (Fig. 4).

## Strengths and challenges of the PLRH model

The PLRH affords patients the ability to conduct research independently, free from the constraints of research strategy and interests that form an inextricable part of most industry or academic research programmes. This broad ethos enables patients to pursue research questions that matter to them in a rigorous and unbiased manner. To our knowledge, this is the first initiative of its kind globally.

The PLRH approach has several strengths: it is the only initiative to retain patient proposers as the drivers of research from the very outset; and, given that proposals originate with patient organisations, patients feel a greater sense of partnership, leading to more rapid recruitment, better participant adherence, and stronger support of studies.

There are also challenges inherent to the PLRH model. Projects often commence from a standing start, without pre-existing literature reviews or meta-analyses. This requires feasibility work which can be time and resource intensive; such early work typically precedes funding applications, meaning that supporting resources need to be raised separately. The nature of proposals is unpredictable, and although generic methodology is provided by the PLRH and CCTU, highly specialised disease areas require support and leadership from experts in a given field. While some investigators are very interested and can provide capacity, others are unable to assist pro bono, and still others view the PLRH with a considerable degree of scepticism and decline to support its activities. Secondly, core infrastructure and staffing are required to maintain the PLRH. Since the initiative is both new and unprecedented, the remit of existing funding streams does not easily align with the focus of the PLRH. Finally, it is clear that the PLRH approach is not suited to all aspects of clinical research. New drugs or devices will naturally emerge from industry or academic research, and it is not reasonable to expect that all highly technical studies should originate with patients.

## Advancing the PLRH model

The PLRH is unique in remit and operation. The recently published NIHR INVOLVE guidance of research co-production cites the key co-production principles as sharing of power, inclusion of perspectives, respecting the value and knowledge of all contributors, reciprocity, and the building of relationships [4]. The PLRH model does more than align with these principles: it surrenders power, is driven by patient perspectives with researchers rather than patients as reciprocators, and maintains the relationship with patient partners as both the over-arching and underpinning principle. Existing models and guidelines for co-produced research focus on recognising patients and the public as equal partners within the historical paradigm of academic or industry-driven research. In contrast, we propose a model that gives ‘power to the people’, with researchers acting as servants and agents of a patient-dictated research agenda.

Our early experience suggests that the PLRH model addresses a need that is currently at best incompletely met across the PPI landscape in the United Kingdom. As we have not yet extensively advertised the PLRH, it is likely that our report significantly underestimates the need for this model of patient led research. There is an urgent requirement to expand the capacity of the PLRH, and indeed to replicate the PLRH model elsewhere. How this is best achieved is the subject of ongoing discussions between the PLRH, INVOLVE, UKCRC, East of England RDS, regional research and involvement groups, and international groups such as PCORI, Canadian research centres, and the International Clinical Trial Center Network. National and international conference presentations on our methods and early success have generated considerable interest and support.

## Abbreviations

ADPKD: Autosomal Dominant Polycystic Kidney Disease
CCTU: Cambridge Clinical Trials Unit
CUHP: Cambridge University Health Partners
EUPATI: European Patients’ Academy
JLA: James Lind Alliance
NCRI: National Cancer Research Institute
NIHR: National Institute for Health Research
PCORI: Patient-Centered Outcomes Research Institute
PKD: Polycystic Kidney Disease
PLRH: Patient Led Research Hub
PPI: Patient and Public Involvement
RDS: Research Design Service
SPOR: Strategy for Patient-Oriented Research
UKCRC: United Kingdom Clinical Research Collaboration
